# Supplementary material for: Comprehensive Annotation and Functional Exploration of MicroRNAs in Lettuce
Source: Front Plant Sci. 2021 Dec 24;12:781836. doi: 10.3389/fpls.2021.781836 (PMC8739914; doi:10.3389/fpls.2021.781836)
Supplement: Supplementary file 2 [file Data_Sheet_8.ZIP › GSM2230749.plot/Lsa-miR393c_Lsat_1_v5_gn_1_126900.1_2029_TPlot.pdf]

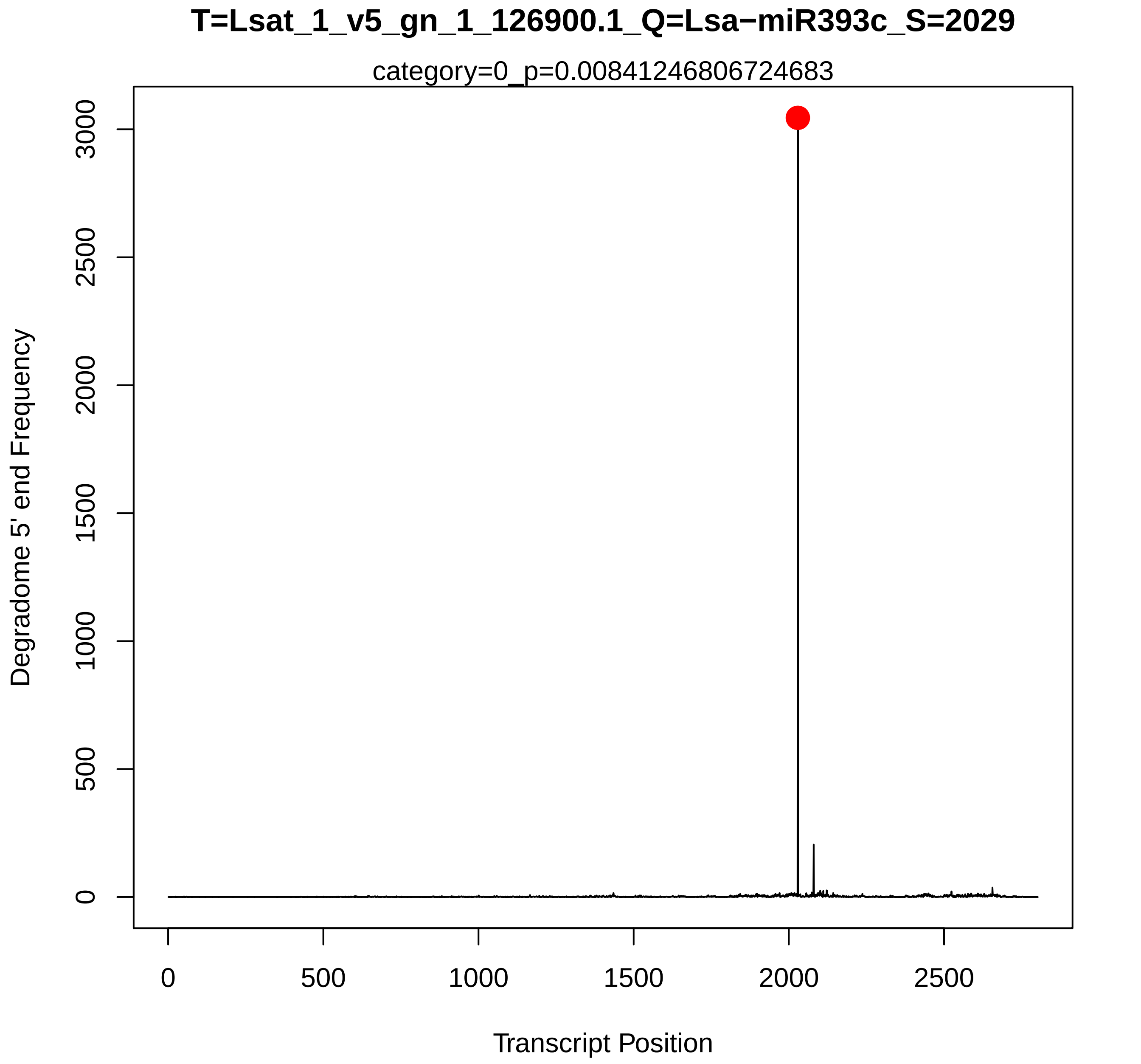
T=Lsat_1_v5_gn_1_126900.1_Q=Lsa−miR393c_S=2029
category=0_p=0.00841246806724683
Degradome 5' end Frequency
0
500
1000
1500
2000
2500
3000
Transcript Position
0
500
1000
1500
2000
2500
